# Tumor response as defined by iRECIST in gastrointestinal malignancies treated with PD-1 and PD-L1 inhibitors and correlation with survival

**DOI:** 10.1186/s12885-021-08944-9

**Published:** 2021-11-19

**Authors:** Peiyi Xie, Hong Zheng, Haiyang Chen, Kaikai Wei, Ximin Pan, Qinmei Xu, Yongchen Wang, Changguan Tang, Olivier Gevaert, Xiaochun Meng

**Affiliations:** 1 Department of Radiology, The Sixth Affiliated Hospital of Sun Yat-sen University, No.26 Yuancunerheng Road, Guangzhou, 510655 Guangdong China; 2 Department of Medicine and Department of Biomedical Data Science, The Stanford Center for Biomedical Informatics Research (BMIR), 1265 Welch Rd, Stanford, CA 94305 USA; 3 Department of Radiation Oncology, The Sixth Affiliated Hospital of Sun Yat-sen University, No.26 Yuancunerheng Road, Guangzhou, 510655 Guangdong China; 4 Department of Medical Imaging, Jinling Hospital, Nanjing University School of Medicine, No.305, Zhongshan East Road, Nanjing, 210002 China

**Keywords:** PD-1/PD-L1 inhibitor, Immunotherapy, Advanced gastrointestinal malignancies, Tumor response, Pseudoprogression

## Abstract

**Background:**

Atypical tumor response patterns during immune checkpoint inhibitor therapy pose a challenge to clinicians and investigators in immuno-oncology practice. This study evaluated tumor burden dynamics to identify imaging biomarkers for treatment response and overall survival (OS) in advanced gastrointestinal malignancies treated with PD-1/PD-L1 inhibitors.

**Methods:**

This retrospective study enrolled a total of 198 target lesions in 75 patients with advanced gastrointestinal malignancies treated with PD-1/PD-L1 inhibitors between January 2017 and March 2021. Tumor diameter changes as defined by immunotherapy Response Evaluation Criteria in Solid Tumors (iRECIST) were studied to determine treatment response and association with OS.

**Results:**

Based on the best overall response, the tumor diameter ranged from − 100 to + 135.3% (median: − 9.6%). The overall response rate was 32.0% (24/75), and the rate of durable disease control for at least 6 months was 30.7% (23/75, one (iCR, immune complete response) or 20 iPR (immune partial response), or 2iSD (immune stable disease). Using univariate analysis, patients with a tumor diameter maintaining a < 20% increase (48/75, 64.0%) from baseline had longer OS than those with ≥20% increase (27/75, 36.0%) and, a reduced risk of death (median OS: 80 months vs. 48 months, HR = 0.22, *P* = 0.034). The differences in age (HR = 1.09, *P* = 0.01), combined surgery (HR = 0.15, *P* = 0.01) and cancer type (HR = 0.23, *P* = 0.001) were significant. In multivariable analysis, patients with a tumor diameter with a < 20% increase had notably reduced hazards of death (HR = 0.15, *P* = 0.01) after adjusting for age, combined surgery, KRAS status, cancer type, mismatch repair (MMR) status, treatment course and cancer differentiation. Two patients (2.7%) showed pseudoprogression.

**Conclusions:**

Tumor diameter with a < 20% increase from baseline during therapy in gastrointestinal malignancies was associated with therapeutic benefit and longer OS and may serve as a practical imaging marker for treatment response, clinical outcome and treatment decision making.

**Supplementary Information:**

The online version contains supplementary material available at 10.1186/s12885-021-08944-9.

## Background

Gastrointestinal cancers are currently one of the most common malignancies in the world [1],and more than 70% of patients with gastrointestinal malignancies have metastases and a poor prognosis [2]. In recent years, immunotherapy has become a major breakthrough in cancer therapy [3] . For advanced or metastatic colorectal cancer (CRC),which is also developing rapidly [3, 4]. Immune checkpoint inhibitors, such as programmed death 1 (PD-1) and programmed death-ligand 1 (PD-L1) antibodies, appear to be one of the most promising approaches in tumor immunotherapy [5, 6]. Compared to conventional cancer therapy, the anticancer mechanism of immune-checkpoint inhibitors is based on the blockade of immune inhibition by tumors, which leads to the simulation of host immunity against tumors, rather than direct cytotoxic or targeted effects to tumor cells [7–9]. In-depth mechanistic studies have shown that T cells play a major role in immune-mediated processes in cancer patients. Moreover, for PD-1/PD-L1 therapy, patterns of tumor response may differ from those observed with chemotherapy or targeted therapy. Atypical tumor response patterns such as “pseudoprogression” were recognized in immune-related responses [7]. Pseudoprogression is defined as an increase in the sum of the longest diameter (SLD) of the target lesions by at least 20% from the baseline after initial immunotherapy followed by a decrease by more than 30% from the time of determination of disease progression, not from baseline [7, 10].

According to the revised Response Evaluation Criteria in Solid Tumors (RECIST 1.1), pseudoprogression is normally categorized as progressive disease (PD) [11]; however, during subsequent follow-up imaging, the tumor burden often actually decreases. This phenomenon poses a challenge to clinicians and investigators, which may lead to misclassifying pseudoprogression as PD and terminating PD-1/PD-L1 inhibitor therapy. The mechanism of pseudoprogression is thought to be caused by the infiltration of T cells into the tumor, leading to a significant increase in the initial tumor burden, rather than the true proliferation of tumor cells [12–14]. Therefore, updated criteria have to be used to capture these atypical response patterns in patients treated with cancer immunotherapy to distinguish them from those of traditional cytotoxic chemotherapy and molecular targeted therapy. Therefore, the most commonly used and validated treatment response criteria of conventional chemotherapies in solid tumors, RECIST 1.1 are not capable of properly assessing response after PD-1/PD-L1 inhibitor therapy [15]. To further improve imaging markers for evaluating the efficacy of PD-1/PD-L1 inhibitor therapy, the consensus guidelines for iRECIST were revised in 2017 [16]. The definition of RECIST 1.1 forms the basis of iRECIST, so the definitions of measurable and nonmeasurable lesions, as well as target and nontarget lesions, remain unchanged. Immune unconfirmed progressive disease (iUPD) has been newly added, and mainly includes the following aspects: first, the assessment of tumor response may be delayed, so two consecutives (time interval of at least 4 weeks) imaging assessments of progressive disease or tumor response evaluation are required; second, the appearance of new lesions does not necessarily indicate the progression in immunotherapy patients. To assess tumor response after PD-1/PD-L1 inhibitor therapy, follow-up imaging studies should be performed at least 4 weeks later to evaluate new lesions [16].

Previous studies lacked detailed immune-related response assessments and tumor diameter changes by iRECIST in advanced gastrointestinal malignancies during PD-1/PD-L1 inhibitor therapy. Whether the continuation of immune checkpoint inhibitor therapy is beneficial, how to evaluate the tumor response during immunotherapy and how to predict overall survival (OS) in advanced gastrointestinal malignancies are currently unclear clinically. The purpose of this study was to systematically analyze the dynamic changes in tumor diameter based on the iRECIST criteria on contrast-enhanced CT (CE-CT) images from baseline scans and follow-up scans during PD-1/PD-L1 inhibitor therapy and to identify imaging biomarkers for tumor response and OS in advanced gastrointestinal malignancies.

## Methods

### Patient selection

**Fig. 1:**
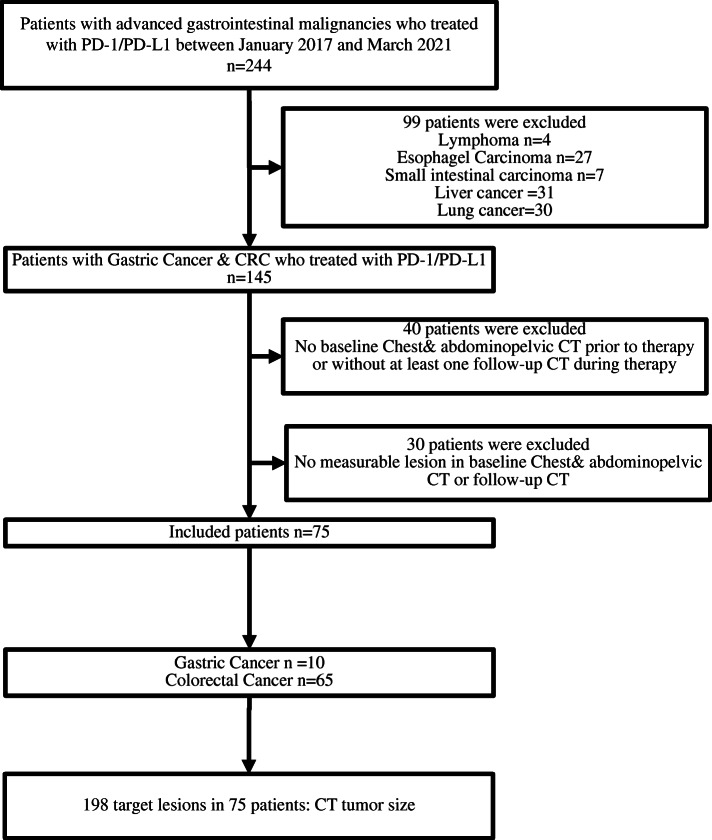
Study Flowchart shows patient enrollment, with the inclusion and exclusion criteria

This retrospective study was approved by the ethics committee of the Sixth Affiliated Hospital of Sun Yat-sen University (Guangzhou, China) and the requirement for informed patient consent was waived. Patients with advanced gastrointestinal malignancies treated with PD-1/PD-L1 inhibitors at the Sixth Affiliated Hospital of Sun Yat-sen University from January 2017 to March 2021 were enrolled. The inclusion criteria were patients who had pathologically proven gastric cancer or colorectal cancer (CRC) and who hadreceived PD1/PD-L1 inhibitor treatment. The exclusion criteria were patients who had a history of malignancy other than gastric cancer or CRC, did not have baseline computed tomography (CT) scans and at least one follow-up CT scan during therapy for review, and did not have at least one measurable lesion at baseline and follow-up CT scans. The patient enrollment process is shown in Fig. 1. Ultimately, 75/244 patients were enrolled. Among the 75 patients (mean age, 48.8 ± 14.1 years), there were 48 men (64.0%) and 27 women (36.0%). The PD-1/PD-L1 inhibitors used clinically included Pembrolizumab, Nivolumab, Toripalimab and Atezolizumab.

### CT techniques

In this study, contrast-enhanced computed tomography (CE-CT) scans were performed on an Aquilion ONE 320-slice scanner (Toshiba, Japan). The scan range was from the apex of the lung to 1 cm below the pubic symphysis. The contrast medium (Ultravist 370: 1.5 mL/kg body weight) was injected at 3.0 mL/s. The threshold CT value of arterial phase scan was set to 180 ~ 200 HU; the portal vein phase was delayed 65–75 s; and the delay period was delayed 180 s. The scanning parameters were as follows: tube voltage,120 kV; automatic tube current; layer thickness, 3 mm; layer interval, 3 mm; rotation time, 0.5 s. Multi-planar reconstruction (MPR) was used to reconstruct the coronary images of the arterial phase and the portal phase. The reconstructed layer thickness was 3 mm and the interval was 3 mm. The CT scan image and the reconstructed image were transmitted to the Picture Archiving Communication System (PACS) workstation for CT line measurement.

### CT tumor diameter measurements

All target lesions at baseline and all follow-up CT images during therapy were blinded and measured by three board-certified radiologists with 5,10 and 15 years of experience in cancer imaging using Immune RECIST (iRECIST) based on previous studies [16–18]. According to iRECIST, target lesions were defined as non-nodal lesions (≥10 mm in the longest diameter) and nodal lesions (≥15 mm in the short axis). Up to two target lesions per organ were tracked and up to 5 total lesions were tracked in total in each patient. Target lesions were measured at baseline and throughout all follow-up CT scans during therapy. The total tumor diameter was measured as the sum of the longest diameter of the non-nodal lesions and the short axis of nodal lesions. The absolute change in the tumor diameter in each patient, as well as the rate of change of the patient in the baseline CT scan and all follow-up CT scans, were calculated.

### Assessment of tumor response and outcome

According to iRECIST [16], the immune best overall response (iBOR) was calculated, using the thresholds of ≥30% decrease compared to baseline for immune partial response (iPR) and ≥ 20% increase compared to nadir of the sum of target lesions with a minimum of 5 mm for immune unconfirmed progressive disease (iUPD). However, iUPD cases must be followed up for confirmation after 4–8 weeks. If the lesion is enlarged (increased by at least 5 mm) or a new lesion appears, immune confirmed progressive disease (iCPD) is confirmed. If the lesion is reduced (compared to baseline), it may be classified as either immune complete response (iCR) or partial response (iPR), or stable disease (iSD). If iUPD reappears, the evaluation standard needs to be reset (compared to the nadir value) and then re-evaluated for iCPD compliance at the next follow-up.

### Statistical analysis

Comparisons across different groups of progression and response patterns were carried out by using a Student’s t test for continuous variables and Fisher’s exact test for categorical variables. The Kaplan–Meier method was used to assess survival outcomes, and the log-rank test was adopted to evaluate the difference between the groups. Univariate Cox models were applied to evaluate associations between OS and the covariates. Multivariable Cox models were used to adjust for clinical variables and potential covariates. According to clinical experience, sex, age, clinical TNM staging, number of prior treatments, cancer type, combined surgery, treatment courses of PD-1/PD-L1 inhibitors, mismatch repair status, histologic grade, histologic types, pathologic TNM staging, lymphovascular invasion, perineural invasion (PNI), PD1/PD-L1 expression, BRAF status, KRAS status were potential variables for consideration. After the univariate Cox analysis, we included age, histologic grade, cancer type, combined surgery into the multivariable analysis (*P* < 0.05). The multivariable analysis showed that histologic grade (HR = 4.55, *P* = 0.1), and cancer type (HR = 0.15, *P* = 0.096) had stronger prognostic roles than combined surgery (HR = 0.24, *P* = 0.19). All *P* values were based on a two-sided hypothesis and a *P* value < 0.05 was considered to be statistically significant.

## Results

### Overview of the patients and target lesions

The clinical characteristics of 75 patients with gastric cancer or CRC are shown in Table 1. These patients, comprised 48 men (64.0%) and 27 women (36.0%) with a mean age of 48.8 ± 14.1 years. The mean BMI was 27.5 ± 11.2. The mean levels of CEA and CA199 pre-immunotherapy were about 160.2 ± 411.3 ng/ml, 387.3 ± 1271.7 ng/ml and the levels of CEA and CA199 post-immunotherapy were about 284.4 ± 890.5 ng/ml, 512.8 ± 2037.1 ng/ml. As shown in Table 2, the clinical cancer staging of 36 patients (48%) was T1–3, and that of 24 (32%) patients was T4. In regard to N staging, N0 was found in 11 patients (14%), N1 in 21 patients (28%), and N2 in 27 patients (36%). M0 staging was found in 23 patients (30%), and M1 staging was found in 38 patients (50%). Seventy-three (97.3%) patients were treated with immunotherapy combined with chemotherapy, 13 (17.3%) were treated with immunotherapy combined radiotherapy, 49 (65.3%) were treated with immunotherapy combined with targeted therapy, and 53 (70.7%) were treated with immunotherapy combined with surgery.

**Table 1:** The Clinical Characteristics of the 75 patients

Parameter		Value
**Gender-no.(%)**	No. of Male(%)	48 (64.0%)
	No.of Female(%)	27 (36.0%)
**Mean age-mean ± SD (yr)**	All subjects	48.8 ± 14.1
	Male	49.0 ± 13.5
	Female	48.4 ± 15.4
**Body mass index-mean ± SD (kg/m**^**2**^**)**		27.5 ± 11.2
**ECOG performance status-no.(%)**	0	18 (24.0%)
	1	57 (76.0%)
**CEA Level-mean ± SD (ng/ml)**	Pre-Immunotherapy	160.2 ± 411.3
	Post-Immunotherapy	284.4 ± 890.5
**CA199 Level-mean ± SD (ng/ml)**	Pre-Immunotherapy	387.3 ± 1271.7
	Post-Immunotherapy	512.8 ± 2037.1
**Previous therapies-no.(%)**	Surgery	53 (70.7%)
	Chemotherapy	73 (97.3%)
	Radiotherapy	13 (17.3%)
	Target cancer therapy	49 (65.3%)

**Table 2:** Patients’ Characteristics in 75 patients

Parameter	Total (%)(***N*** = 75)	Typical response (%)(***N*** = 24)	Typical progression (%)(***N*** = 15)	iUPD (%)(***N*** = 9)	Stable Disease (%)(***N*** = 25)	Pseudoprogression (%)(***N*** = 2)	***P*** Value
**Gender**							0.06
Male	48 (64)	12 (50)	10 (66)	9 (100)	15 (60)	2 (100)	
Female	27 (36)	12 (50)	5 (33)	0	10 (40)	0	
**Mean age ± SD, y**	48.8 ± 14.15	46.2 ± 15.6	48.3 ± 16.27	56.3 ± 8.18	48.84 ± 12.75	49 ± 19.79	0.51
**Clinical T stage (cT)**							0.28
T1–3	36 (48)	13 (54)	3 (20)	5 (55)	14 (56)	1 (50)	
T4	24 (32)	7 (29)	8 (53)	4 (44)	4 (16)	1 (50)	
Not investigated	15 (20)	4 (16)	4 (26)	0	7 (28)	0	
**Clinical N stage (cN)**							0.98
0	11 (14)	4 (16)	2 (13)	3 (33)	2 (8)	0	
N1	21 (28)	8 (33)	5 (33)	2 (22)	4 (16)	2 (100)	
N2	27 (36)	8 (33)	4 (26)	4 (44)	11 (44)	0	
Not investigated	16 (21)	4 (16)	4 (26)	0	8 (32)	0	
**Clinical M stage (cM)**							0.94
0	23 (30)	7 (29)	2 (13)	4 (44)	9 (36)	1 (50)	
1	38 (50)	13 (54)	9 (60)	5 (55)	10 (40)	1 (50)	
Not investigated	14 (18)	4 (16)	4 (26)	0	6 (24)	0	
**Mean treatment course ± SD**							0.007
	8.6 ± 7.3	12.2 ± 8.9	8.7 ± 4.5	3.0 ± 1.2	6.8 ± 6.6	11.5 ± 9.2	
**Mismatch Repair Status**							0.04
Mismatch Repair-Proficient (pMMR)	41 (54)	12 (50)	7 (46)	9 (100)	12 (48)	1 (50)	
Mismatch Repair-Deficient (dMMR)	22 (29)	9 (37)	2 (13)	0	10 (40)	1 (50)	
Not investigated	12 (16)	3 (12)	6 (40)	0	3 (12)	0	
**Histologic types**							0.72
Adenocarcinoma	58 (77)	20 (83)	9 (60)	9 (100)	18 (72)	2 (100)	
Mucinous adenocarcinoma	8 (10)	2 (8)	2 (13)	0	4 (16)	0	
Signet ring cell carcinoma	1 (1)	0	1 (6)	0	0	0	
Other	8 (10)	2 (8)	3 (20)	0	3 (12)	0	
**KRAS status**							0.54
Wild-type	21 (28)	6 (25)	3 (20)	4 (44)	7 (28)	1 (50)	
Mutated	16 (21)	6 (25)	2 (13)	1 (11)	7 (28)	0	
Not investigated	38 (50)	12 (50)	10 (66)	4 (44)	11 (44)	1 (50)	

Next, using the iRECIST criteria, a total of 198 target lesions in 75 patients were analyzed. There were 68 (34.3%) abdominal and pelvic implant lesions, 60 (30.3%) liver lesions, 20 (10.1%) lung lesions, 48 (24.2%) node lesions, one (0.5%) spleen lesion and one (0.5%) adrenal lesion. The median in each patient was 2.6 lesions (range: 1–5). The median baseline diameter of 198 measurable lesions was 2 mm (range: 10–127 mm).

### Tumor diameter changes and immune-related responses

**Fig. 2:**
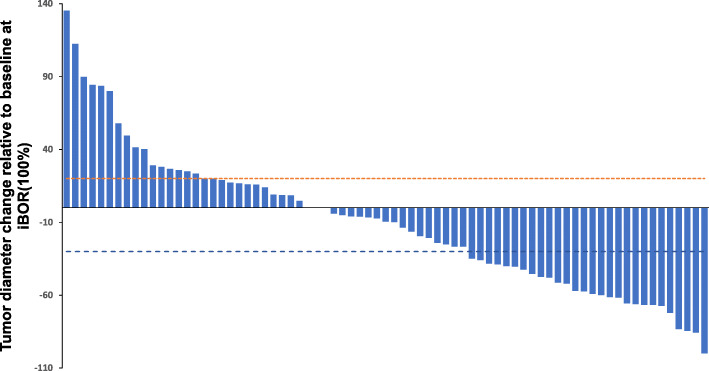
A waterfall plot of the tumor diameter change of target lesions at iBOR from baseline in 75 patients with advanced gastrointestinal tumors with measurable lesions. Dashed lines of + 20% and − 30% indicate thresholds for immune progressive disease (iPD) and immune partial response (iPR), and each bar represents a patient

The median follow-up for 75 patients was 24.7 months after initial immunotherapy. The immune best overall response (iBOR) was the best timepoint response recorded from the start to the end of immunotherapy. Tumor diameter changes compared to baseline at iBOR in 75 patients ranged from − 100 to + 135.3% (median: − 9.6%). Figure 2 shows the details of iBOR in 75 patients. According to iRECIST and iBOR [10], we defined a typical response as complete response or partial response and defined typical progression as progressive disease after confirmation by 4–8 weeks follow-up (iCPD). Immune unconfirmed progressive disease without follow-up after 4–8 weeks was denoted as iUPD. The classification of the 75 patients in this study based on their response to immunotherapy is reported in Table 2 and Table S1 in Supplemental materials.

A total of 24 patients had a typical response (32.0%,23 iPR, one iCR), 15 patients had typical progression (20.0%), 9 had iUPD (12.0%), 25 had stable disease (33.3%), and 2 had pseudoprogression (2.7%). In this study, in the 75 advanced gastrointestinal cancer patients, the overall response rate was 32.0% (24/75). The definition of durable disease control was a less than 20% increase in tumor diameter compared to baseline, which lasted for at least 6 months. In this study, durable disease control was noted in 23 patients (23/75,30.7%). Among these 23 patients, iBOR was iPR in 20 patients, iCR in one patient and iSD in 2 patients. The differences in the PD-1/PD-L1 treatment course in the typical response, typical progression, iUPD, stable disease and pseudoprogression groups were statistically significant (*P* = 0.007). Moreover, the differences in pMMR and dMMR in the response, progression, iUPD, stable disease and pseudoprogression groups were statistically significant (*P* = 0.04).

### Association between tumor diameter dynamics and overall survival

**Fig. 3:**
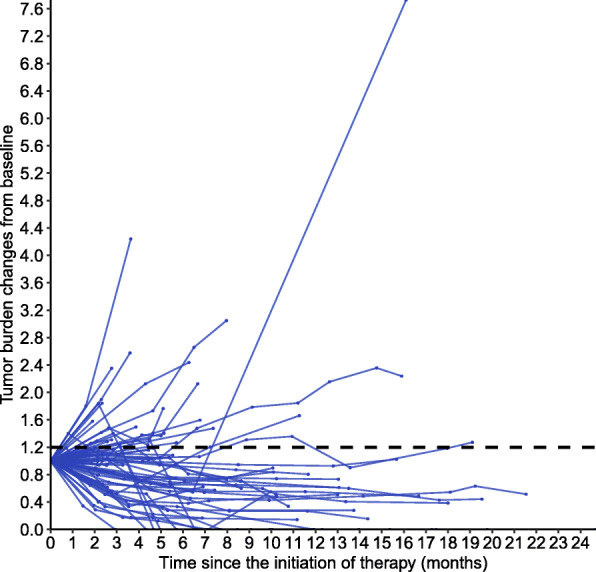
Spider plot of tumor diameter dynamics during PD-1/PD-L1 inhibitor therapy in 75 patients with measurable lesions. During the follow-up, patients with tumor diameter that maintained a < 20% increase from baseline were classified as the treatment benefit group (*n* = 48; tumor diameter maintained a < 20% increase in those patients)

The median follow-up time of the 75 patients was 24.0 months (range: 3.6–79.6 months), and 10 (13.3%) deaths were observed among these patients throughout follow-up. Next, we used spider plots to demonstrate tumor diameter dynamics throughout PD-1/PD-L1 inhibitor therapy in all 75 patients (Fig. 3). Forty-eight patients had the tumor diameters maintaining a < 20% increase compared to baseline. These patients were grouped into the < 20% group (48/75, 64.0%). Twenty-seven patients had a tumor diameter increase ≥20% compared to baseline at some time point throughout PD-1/PD-L1 inhibitor therapy. These patients were grouped into the ≥20% group (27/75, 36.0%). Among these patients, two patients demonstrated an atypical response pattern defined as pseudoprogression. By observing the spider plot for tumor diameter dynamics, we compared the relationship between OS and the subgroups defined by the threshold of 20% increase from baseline.

**Fig. 4:**
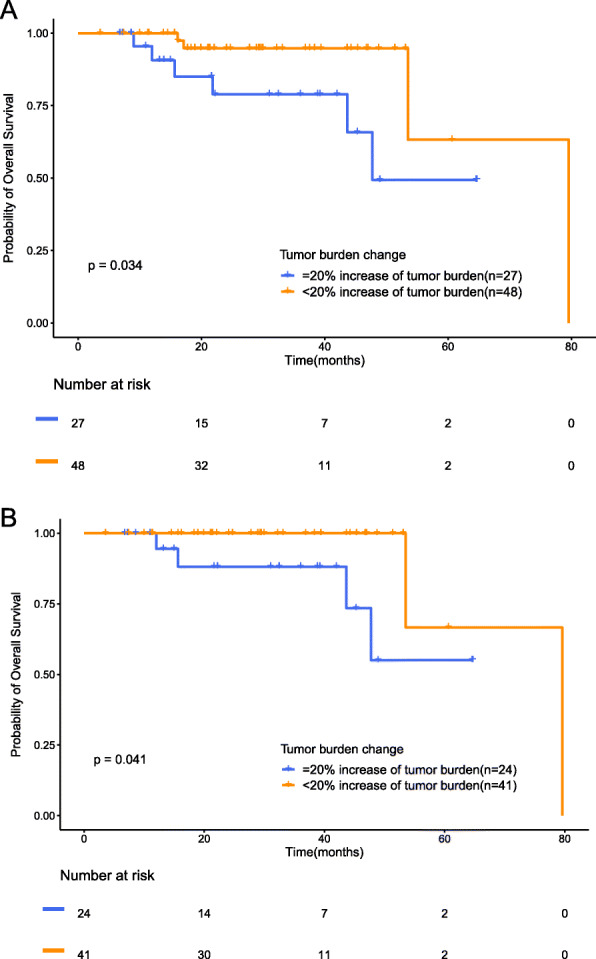
Kaplan–Meier analysis of changes in OS and tumor diameter in patients. Compared with baseline, the OS in the tumor diameter increase of the < 20% group was longer than that in the tumor diameter increase of the ≥20% group. **(A)** Overall survival in the cohorts of advanced gastrointestinal cancer patients. **(B)** Overall survival of patients with colorectal cancer

Using Kaplan–Meier analysis, the < 20% group had a longer OS than the ≥20% group (Fig. 4a, median OS: 80 months vs. 48 months, HR = 0.22, log-rank *P* = 0.034). Patients with poor cancer differentiation had higher hazards of death than patients with well to moderate cancer differentiation (HR = 2.04, log-rank *P* = 0.06). Combined surgery had a lower hazard of death (HR = 0.15, log-rank *P* = 0.01). We also found significant differences for age (HR = 1.09, log-rank *P* = 0.01) and cancer type (HR = 0.23, log-rank *P* = 0.001). However, there were no difference in treatment course (HR = 0.84, log-rank *P* = 0.09), KRAS status (HR = 2.43, log-rank *P* = 0.09), sex (HR = 1.02, log-rank *P* = 0.97) or MMR (HR = 1.04, log-rank *P* = 0.92). In the multivariable analysis, the 48 patients with tumor diameters that maintained a < 20% increase from baseline had significantly reduced hazards of death (HR = 0.15, *P* = 0.01) after adjusting for age, combined surgery, KRAS status, cancer type, MMR, treatment course and cancer differentiation. Based on the above subgrouping method, for the cohort of 65 colorectal cancer patients, 41 patients were grouped into the < 20% group, and the remaining 24 patients were grouped into the ≥20% group. According to univariate analysis, patients in the < 20% group had a longer OS than those in the ≥20% group, and a reduced risk of death (Fig. 4b, median OS: 80 months vs. undefined, HR = 0.15, *P* = 0.041 by the log-rank test). For the cohort of 10 GC patients, 7 patients were grouped into the < 20% group, and the remaining 3 patients were grouped into the ≥20% group, which had a reduced risk of death (median OS: undefined vs. 21.8 months, HR = 3.00, *P* = 0.242 by the log-rank test) (Fig.S1 in Supplemental materials).

### Atypical response pattern

**Fig. 5:**
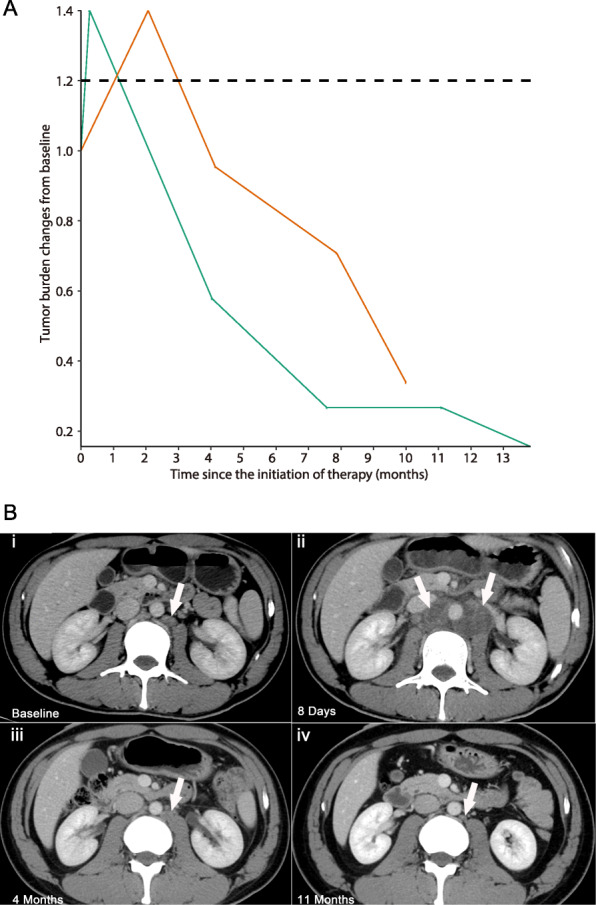
Patients with measurable lesions showing pseudoprogression in terms of tumor diameter. (**A**) The spider plot of tumor diameter changes showing 2 patients with pseudoprogression. The tumor diameter of both 2 patients increased by more than 40% from baseline and then gradually decreased in the subsequent follow-up. The tumor diameter decreased by more than 30% compared to the peak. (**B**) A 35-year-old male with advanced rectal cancer with pseudoprogression, corresponding to the green line in **(A**). A baseline CT scan showed the measuring 10 mm in the short diameter of the left aortic lymph nodes of the abdominal aorta (**i**, arrow). On the 1st day and at 8 days, the diameter of the lesion (36 mm) increased significantly. A newly enlarged lymph node on the right side of the abdominal aorta, with a short diameter measuring 20 mm (**ii**); 2nd follow-up scans at 4 months (**iii**) showing that the left aortic lymph nodes of the abdominal aorta were significantly reduced (11 mm). The abdominal aortic right para-lymph node disappeared. At the 3rd follow-up scan at 11 months (**iv**), the left aortic lymph nodes of the abdominal aorta were still smaller than before, and the measured value of the short diameter was < 5 mm. Since then, the left aortic lymph nodes of the abdominal aorta still had a short diameter of < 5 mm and a maintained durable response

In this study, 2 patients (2.7%) demonstrated pseudoprogression. After the initial PD-1/PD-L1 inhibitor therapy, the tumor diameter of both patients increased by 40% compared to baseline, and new lesions appeared in the first follow-up. During subsequent follow-up, the tumor diameter gradually decreased. Compared with the peak tumor diameter, both lesions were reduced by more than 30%, as shown in Fig. 5a and b.

## Discussion

Gastrointestinal malignancies are one of the most common tumors, and their incidence and mortality have been increasing in recent years [19], especially colorectal cancer (CRC), which is the third most common cancer and the second in terms of mortality [19]. The treatment of CRC includes surgery, chemotherapy, radiotherapy, and molecular targeted therapy. These treatments have improved the prognosis of patients to some extent. Unlike traditional tumor therapies, tumor immunotherapy works by improving the patient’s immune system and destroying tumor cells through the immune system. Therefore, patients receiving immunotherapy respond relatively slowly. However, emerging immunotherapies, such as PD-1/PD-L1 inhibitor treatment, can show atypical response patterns such as pseudoprogression [4, 7, 20, 21]. Imaging plays an important role in evaluating and characterizing immune-related tumor responses and progression and is becoming increasingly important for clinical treatment decisions [7].

iRECIST is the latest imaging criterion for evaluating the tumor diameter after immunotherapy [16]. Using the iRECIST criteria, this study evaluated tumor diameter dynamics on serial CT scans in 75 patients with advanced gastrointestinal tumors treated with PD-1/PD-L1 inhibitors. We found that patients with a tumor diameter that maintained a < 20% increase compared to baseline measurements throughout therapy had longer OS. Notably, many patients can benefit from PD-1/PD-L1 inhibitor therapy as long as their tumor diameter maintains a < 20% increase from baseline. Therefore, we infer that the threshold of tumor diameter maintained at < 20% increase will provide a potential imaging biomarker of whether patients with advanced gastrointestinal tumors should continue treatment with PD-1/PD-L1 inhibitors, which is expected to play an important role in clinical decision-making.

In this study, 75 patients with gastrointestinal malignancies received PD-1/PD-L1 treatment, and the tumor response rate was 32.0%. The definition of durable disease control is a tumor diameter with a less than 20% increase compared to baseline, which lasts for at least 6 months. In this study, durable disease control was noted in 23 patients (23/75,30.7%). Figure 3 provides the dynamic changes in the tumor burden of the patients in this study during the follow-up, reflecting the information about the immune-related response patterns in the cohort. Using univariate analysis, the < 20% group had a longer OS than the ≥20% group, and this finding is similar to that of previous studies of immunotherapy for melanoma and lung cancer [18, 22].

Next, 48 patients with tumor diameter that maintained a < 20% increase from baseline had significantly reduced hazards of death (HR = 0.15, *P* = 0.01) after adjusting for age, combined surgery, KRAS status, cancer type, MMR, treatment course and cancer differentiation using multivariable analysis. According to previous research [23–25], deficient mismatch repair (dMMR) is a good predictor of the clinical benefit of PD-1/PD-L1 inhibitor therapy in many cancers, especially CRC. Based on MMR status, CRC patients can be divided into two subgroups based on the clinical benefit of immunotherapy: dMMR colorectal cancer (dominant population) and pMMR colorectal cancer (ineffective population). Based on the dynamic changes in the tumor diameter of 75 patients with advanced gastrointestinal tumors, iBOR was obtained for each patient. According to iRECIST, the responses were divided into typical response, typical progression, immune disease progression (iUPD), stable disease and pseudoprogression. The difference in MMR between the above two groups was statistically significant (*P* = 0.04). Moreover, for the cohort of 65 CRC patients, 41 patients who had tumor diameters with less than a 20% increase had a longer OS and a reduced risk of death.

In previous studies [18, 22], immune-related response patterns of tumor burden dynamics have been described for the treatment monitoring of patients with advanced non-small cell lung cancer (NSCLC) and advanced melanoma. However, studies have reported limited data on advanced gastrointestinal tumors [26].

The iRECIST criteria include immune-related response patterns such as the response after initial increase and the appearance of new lesions [16]. These phenomena are often defined as pseudoprogression. Recognizing pseudoprogression is an important challenge when deciding whether to continue treatment after immunotherapy [7]. Previous studies have reported pseudoprogression in patients with advanced melanoma, NSCLC and metastatic gastrointestinal stromal tumors [10, 18, 22, 26]. This study identified two patients with pseudoprogression. One patient had gastric cancer, and the other had colorectal cancer. Studies have shown that the incidence of pseudoprogression is low, in most cases not exceeding 10% [10, 27–29]. In this study, of the 75 patients with advanced gastrointestinal malignancies with measurable lesions, 2(2.7%) patients showed pseudoprogression, and the tumor diameter increased by more than 40% at the first follow-up (8.9 weeks and 1.1 weeks) after immunotherapy. A second follow-up was performed in the fourth month and it was found that the tumor diameter gradually decreased, reaching a more than 30% decrease at 10 months and 4 months after immunotherapy. The results of this study are basically consistent with the conclusions of previous studies; pseudoprogression occurred in most patients within 12 weeks after immunotherapy [30].

This study has the following limitations. First, one of the limitations of this study is that the patients in this study had already received other treatments and the time of hospital admission was different from the time of receiving commercial PD-1/PD-L1 treatment, which will lead to data heterogeneity. Secondly, according to the iRECIST criteria, lesions from the primary site in gastrointestinal cancer patients are not counted in the target or measurable lesions for evaluation. Third, this study reports on a retrospective design from a single institution, most of the patients’ information such as PD1/PD-L1 expression, BRAF status, and KRAS status, was not tested, and that information could not be analyzed well. Thus, future prospective multicenter studies are warranted.

In summary, our study demonstrated that patients with a tumor diameter that maintained a < 20% increase from baseline during PD-1/PD-L1 inhibitor therapy had a therapeutic benefit and longer OS. This factor might be a practical imaging biomarker for treatment response and clinical outcome, and has the potential to play an important role in treatment decisions. The phenomenon of pseudoprogression was uncommon and was observed in only 2 patients (2.7%). The immune-related response of patients with only primary tumors and nonmeasurable lesions requires further study.

## Supplementary Information

**Additional file 1.**

**Additional file 2.**

## Data Availability

The datasets generated during the current study are not publicly available due to limitation of ethical approval involving the patient data and anonymity but are available from the corresponding author upon reasonable request.

## Abbreviations

OS: Overall survival
iRECIST: Immune-related Response Evaluation Criteria in Solid Tumors
iCPD: “immune” confirmed progressive disease
iUPD: “immune” unconfirmed progressive disease
iCR: “immune” complete response
iPR: “immune” partial response
iSD: “immune” stable disease
MMR: Mismatch repair status
dMMR: Mismatch repair-deficient
