# Supplementary material for: Tumor response as defined by iRECIST in gastrointestinal malignancies treated with PD-1 and PD-L1 inhibitors and correlation with survival
Source: BMC Cancer. 2021 Nov 19;21:1246. doi: 10.1186/s12885-021-08944-9 (PMC8605503; doi:10.1186/s12885-021-08944-9)
Supplement: Supplementary file 1 — Additional file 1. [file 12885_2021_8944_MOESM1_ESM.docx]

| **Supplementary materials**  **Table S1 Patients' Characteristics in 75 patients** | | | | | | | |
| --- | --- | --- | --- | --- | --- | --- | --- |
| **Parameter** | **Total**  **(%)(N=75)** | **Typical response (%)(N=24)** | **Typical progression (%)(N=15)** | **iUPD (%)(N=9)** | **Stable Disease (%)(N=25)** | **Pseudoprogression (%)(N=2)** | **P Value** |
| **No.of prior treatments** | | | | | | | 0.51 |
| 1 | 8(10) | 5(20) | 2(13) | 0 | 1(4) | 0 |  |
| 2 | 25(33) | 10(41) | 5(33) | 4(44) | 6(24) | 0 |  |
| 3 | 33(44) | 6(25) | 6(40) | 4(44) | 15(60) | 2(100) |  |
| 4 | 9(12) | 3(12) | 2(13) | 1(11) | 3(12) | 0 |  |
| **Treatment courses of** | | | | | | | 0.14 |
| **PD-1/PD-L1 inhibitors** | | | | | | |  |
| ≤10 | 54(72) | 13(54) | 10(66) | 9(100) | 21(84) | 1(50) |  |
| (10,20] | 16(21) | 7(29) | 5(33) | 0 | 3(12) | 1(50) |  |
| (20,30] | 4(5) | 3(12) | 0 | 0 | 1(4) | 0 |  |
| ＞30 | 1(1) | 1(4) | 0 | 0 | 0 | 0 |  |
| **Histologic grade** |  |  |  |  |  |  | 0.91 |
| Well to moderately differentiated | 41(54) | 14(58) | 8(53) | 5(55) | 13(52) | 1(50) |  |
| Poor differentiated | 23(30) | 7(29) | 4(26) | 3(33) | 8(32) | 1(50) |  |
| Other | 11(14) | 3(12) | 3(20) | 1(11) | 4(16) | 0 |  |
| **Pathologic T stage(pT)** |  |  |  |  |  |  | 0.90 |
| T1-3 | 24(32) | 5(20) | 2(13) | 3(33) | 13(52) | 1(50) |  |
| T4 | 11(14) | 3(12) | 2(13) | 2(22) | 4(16) | 0 |  |
| Not investigated | 40(53) | 16(66) | 11(73) | 4(44) | 8(32) | 1(50) |  |
| **Pathologic N stage(pN)** |  |  |  |  |  |  | 0.20 |
| 0 | 16(21) | 6(25) | 3(20) | 2(22) | 5(20) | 0 |  |
| N1 | 18(24) | 8(33) | 0 | 3(33) | 6(24) | 1(50) |  |
| N2 | 12(16) | 1(4) | 2(13) | 2(22) | 7(28) | 0 |  |
| Not investigated | 29(38) | 9(37) | 10(66) | 2(22) | 7(28) | 1(50) |  |
| **Pathologic M stage(pM)** |  |  |  |  |  |  | 0.23 |
| 0 | 19(25) | 3(12) | 3(20) | 3(33) | 9(36) | 1(50) |  |
| 1 | 43(57) | 17(70) | 7(46) | 4(44) | 14(56) | 1(50) |  |
| Not investigated | 13(17) | 4(16) | 5(33) | 2(22) | 2(8) | 0 |  |
| **Lymphovascular invasion** |  |  |  |  |  |  | 0.88 |
| Negative | 36(48) | 11(45) | 4(26) | 7(77) | 13(52) | 1(50) |  |
| Positive | 10(13) | 3(12) | 2(13) | 0 | 5(20) | 0 |  |
| Not investigated | 29(38) | 10(41) | 9(60) | 2(22) | 7(28) | 1(50) |  |
| **Perineural invasion(PNI)** |  |  |  |  |  |  | 0.37 |
| Negative | 31(41) | 11(45) | 3(20) | 4(44) | 12(48) | 1(50) |  |
| Positive | 15(20) | 3(12) | 3(20) | 3(33) | 6(24) | 0 |  |
| Not investigated | 29(38) | 10(41) | 9(60) | 2(22) | 7(28) | 1(50) |  |
| **PD1/PD-L1 Expression** |  |  |  |  |  |  | 0.54 |
| Negetive | 16(21) | 7(29) | 3(20) | 1(11) | 5(20) | 0 |  |
| Positive | 5(6) | 1(4) | 0 | 1(11) | 3(12) | 0 |  |
| Not investigated | 54(72) | 16(66) | 12(80) | 7(77) | 17(68) | 2(100) |  |
| **BRAF status** |  |  |  |  |  |  | 0.32 |
| Wild-type | 31(41) | 8(33) | 5(33) | 5(55) | 13(52) | 0 |  |
| Mutated | 3(4) | 2(8) | 0 | 0 | 0 | 1(50) |  |
| Not investigated | 41(54) | 14(58) | 10(66) | 4(44) | 12(48) | 1(50) |  |
