# Supplementary material for: Tumor response as defined by iRECIST in gastrointestinal malignancies treated with PD-1 and PD-L1 inhibitors and correlation with survival
Source: BMC Cancer. 2021 Nov 19;21:1246. doi: 10.1186/s12885-021-08944-9 (PMC8605503; doi:10.1186/s12885-021-08944-9)
Supplement: Supplementary file 2 — Additional file 2. [file 12885_2021_8944_MOESM2_ESM.docx]

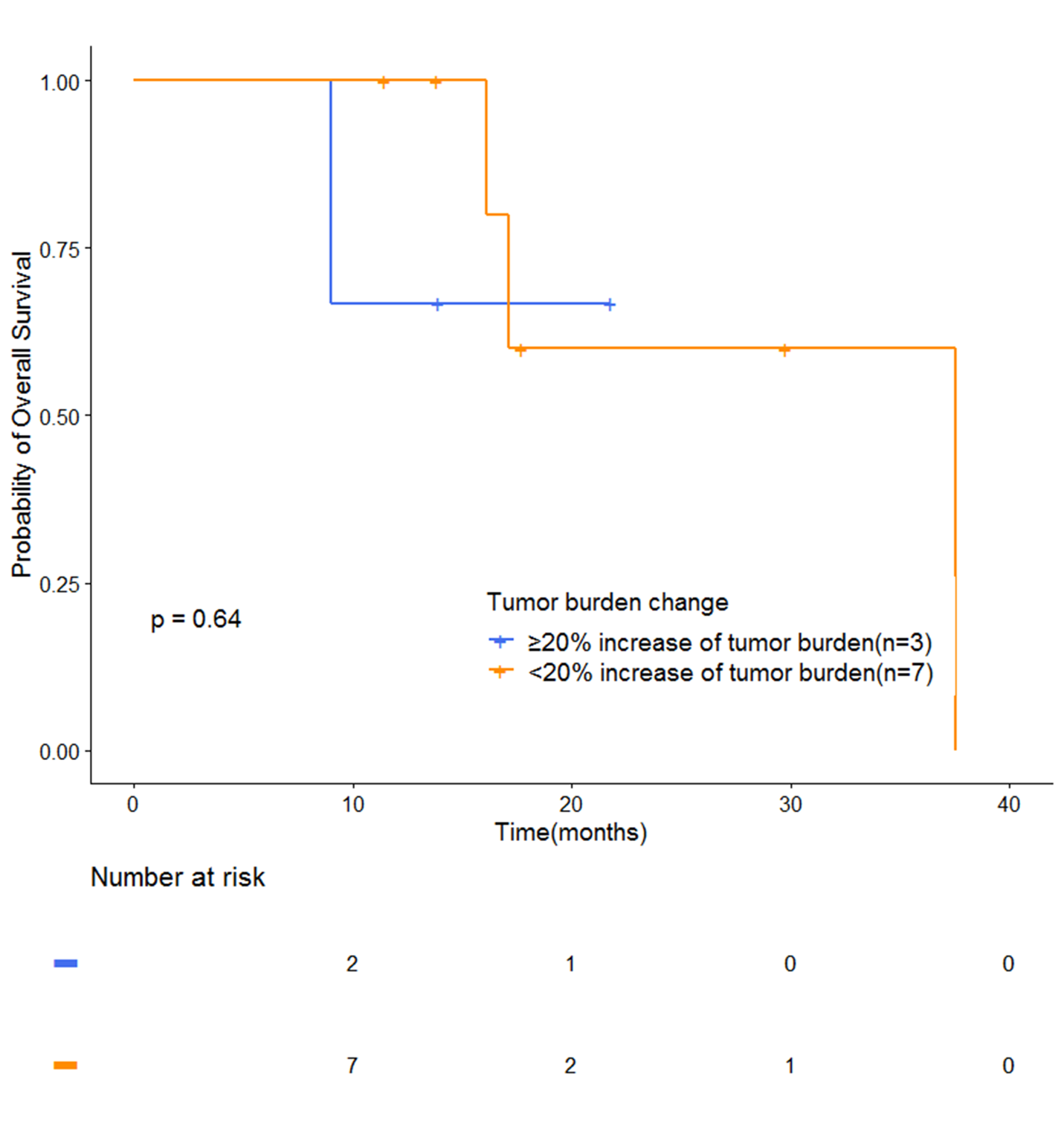


**Figure S1**.   Kaplan-Meier analysis of changes in OS and tumor diameter in patients. Compared with baseline, the OS in the tumor diameter increase of <20% group was longer than that in the tumor diameter increase of the ≥ 20% group. Overall survival in the cohorts with GC patients.
